# Finding the mutations that drive resistance

**DOI:** 10.7554/eLife.57678

**Published:** 2020-05-15

**Authors:** Nadine Bley

**Affiliations:** 1 Department of Molecular Cell Biology, Martin Luther University Halle-Wittenberg Halle Germany; 2 Institute of Molecular Medicine, Martin Luther University Halle-Wittenberg Halle Germany

**Keywords:** variant calling, machine learning, tumor evolution, Human

## Abstract

Mutations that allow tumors to evolve and become resistant to treatment can be readily identified with a new sequencing approach.

**Related research article** Carrami EM, Sharifzadeh S, Wietek NC, Artibani M, El-Sahhar S, Sauka-Spengler T, Yau C, Tresp V, Ahmed AA. 2020. A highly accurate platform for clone-specific mutation discovery enables the study of active mutational processes. *eLife* **9**:e55207. doi: 10.7554/eLife.55207

Despite being a major cause of death, cancer is still far from being fully understood. Most cancer treatments target mutations that happen during the very early stages of the disease, as these genetic variants will be present in the majority of tumor cells (Bailey et al., 2018). However, not all cells inside a tumor are genetically identical, and this heterogeneity is one of the biggest problems in cancer therapy (Gatenby and Brown, 2018). As tumors evolve and become more heterogeneous, some cancer cells acquire new mutations that make them resistant to certain treatments, and drugs targeting these sites could prevent cancers from reoccurring (Figure 1A).

**Figure 1.**
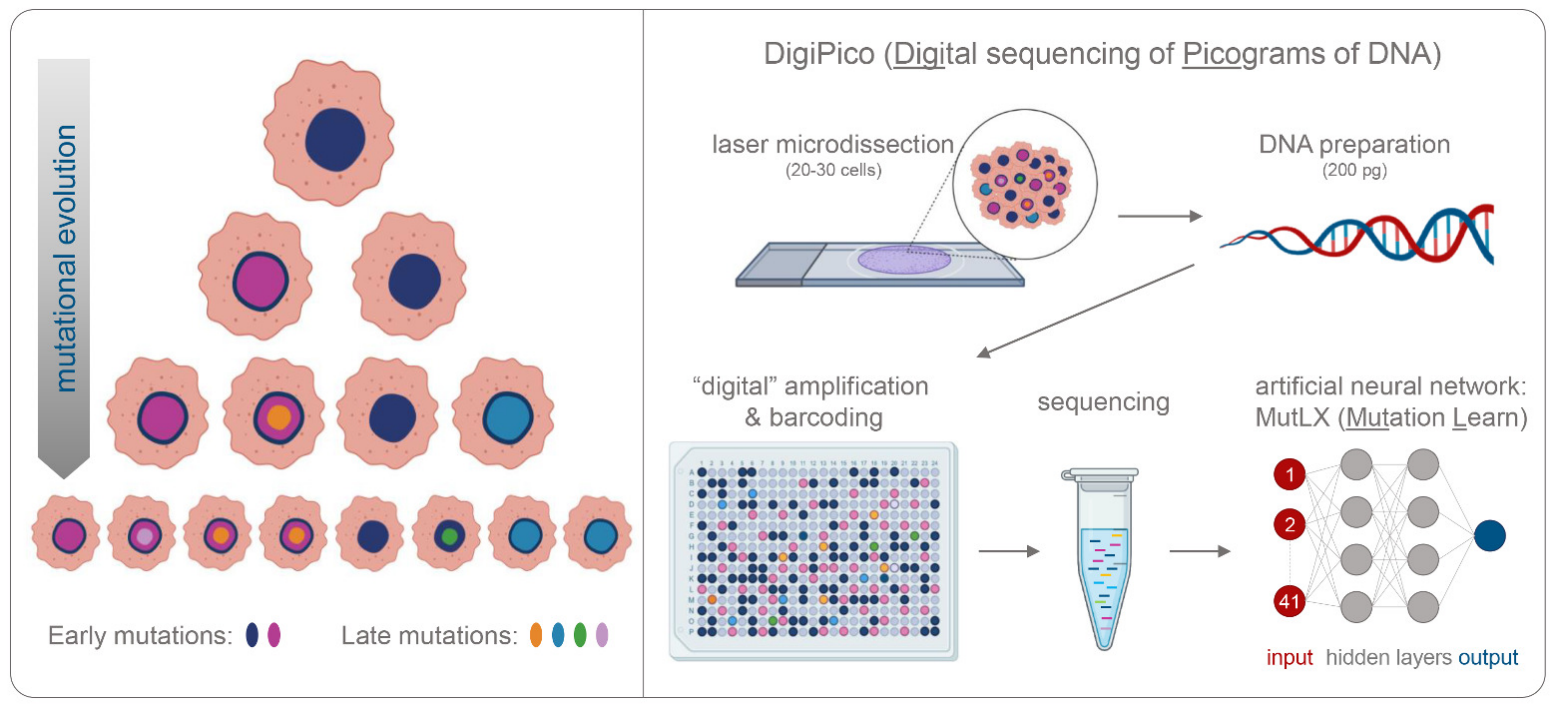
Detecting rare mutations in tumor cells. (**A**) Cancer usually begins with a mutation (dark blue shape in top cell) in a single tumor cell that it passes on to its daughter cells following division. A daughter cell can then gain a new mutation (shown in pink) that it passes on to its progeny. These cells also divide and acquire new mutations (shown in different colors). Over time this leads to a population of cells that are genetically distinct from each other: the initial mutation is present in all the cells, whereas mutations that occurred later are present in a smaller number of cells (bottom row). (**B**) Now, KaramiNejadRanjba et al. have created a sequencing approach called DigiPico that can identify mutations that occur later during tumor evolution. First, cell material is extracted from a small group of 20–30 cells using laser microdissection and diluted down to single molecules of DNA which are plated into 384 individual wells (top panel). The DNA molecule in each well is amplified to create individual libraries, which are then combined and sequenced (bottom panel). After sequencing, an artificial neural network called MutLX is applied to the data to determine which of the genetic variants put into the algorithm (shown in dark red) are mutations that appear later during tumor evolution (shown in dark blue) and which are artefacts generated by the amplification process. Figure created using BioRender (BioRender.com).

Whole-genome sequencing is a widely used strategy for identifying genetic variants which are present in the majority of tumor cells. However, mutations that arise later during tumor evolution are almost impossible to detect using this approach, as they only appear in a handful of cells (Figure 1A; Hrdlickova et al., 2017; Turajlic et al., 2019). Although whole-genome sequencing of single cells can uncover these rarer mutations, this technique requires a high number of individual cells, which are often difficult to collect and expensive to sequence. In addition, amplifying the small quantities of DNA extracted from tumor cells often introduces errors that can be mistaken for cancer mutations. Now, in eLife, Ahmed Ahmed from the University of Oxford and co-workers from the United Kingdom and Germany – including Eli M Carrami as first author – report how they developed a new whole-genome sequencing technique that can overcome these limitations and identify mutations that occur later in tumor evolution (Carrami et al., 2020).

In the first stage of this approach, termed DigiPico, genomic material was extracted from a small group of tumor cells and diluted down to 384 single molecules of DNA (Figure 1B). Each DNA molecule was then individually amplified to create a library of repeated fragments which were sequenced into reads. Carrami et al. hypothesized that if a mutation is randomly generated during the amplification process, it will only appear in a small proportion of the repeated fragments. However, if a mutation is truly related to the cancer sample, it will be present from the start and will therefore be detected in all the reads that result from amplifying the single DNA molecule. Moreover, true mutations that arise during tumor evolution will be distributed regularly across the different libraries, while artefacts that arise during amplification will appear more randomly.

The sequencing data were analyzed using common processing and mapping algorithms and compared to whole-genome sequencing data from the tumor material and blood of the same patient. This identified genomic variants that were unique to the DigiPico data, and true positive mutations that were also found in the majority of tumor cells and the blood of patients. KaramNejadRanjbar et al. then employed a neural network called MutLX, which uses a form of artificial intelligence, to determine whether the unique variants detected by DigiPico were artefacts or cancer related mutations (LeCun et al., 2015). Excluding artefacts dramatically decreased the number of candidates for mutations that occurred late in the evolution of the tumor.

Experimentally validating some of the detected mutants revealed that together, DigiPico and MutlX, are able to eliminate false positives and identify rare mutations. Using the new technique, KaramNejadRanjbar et al. were able to discover a hyper-mutation event called kataegis in a recurring ovarian tumor (Chan and Gordenin, 2015), which conventional sequencing approaches had not been able to detect.

One of the main advantages of this method is that it can identify rare mutations that appear late during tumor evolution from just a small sample of cells. Furthermore, the precision and robustness of the technique also makes it easier to characterize current mutational processes, even in cancers which have a high number of genomic re-arrangements, such as ovarian tumor cells. These findings demonstrate how DigiPico and MutLX can be used to study the evolution of tumors, during cancer development, progression and recurrence.
